# *Rhodotorula benthica* culture as an alternative to antibiotics improves growth performance by improving nutrients digestibility and intestinal morphology, and modulating gut microbiota of weaned piglets

**DOI:** 10.3389/fmicb.2022.964531

**Published:** 2022-09-02

**Authors:** Qianqian Zhang, Jian Li, Xin Yi, Zipeng Li, Shuang Liang, Zhengfeng Fang, Yan Lin, Shengyu Xu, Bin Feng, Yong Zhuo, De Wu, Lianqiang Che

**Affiliations:** ^1^Key Laboratory of Animal Disease-Resistant Nutrition of Sichuan Province, Institute of Animal Nutrition, Sichuan Agricultural University, Chengdu, China; ^2^The First Affiliated Hospital, Department of Pain, Hengyang Medical School, University of South China, Hengyang, China; ^3^Guangzhou Prosyn Biological Technology Feed CO., LTD., Guangzhou, China

**Keywords:** weaning piglets, growth performance, nutrients digestibility, antioxidant property, intestinal morphology, microbiota community

## Abstract

The effects of *Rhodotorula benthica* culture (RBC) and antibiotics (AB) on the growth performance, nutrients digestibility, morphological indicators, and colonic microbiota of weaning piglets were explored. Ninety-six (Duroc × Landrace × Large) weaned piglets (21-day-old) weighing 7.7 ± 0.83 kg, were randomly allocated to 4 dietary treatments. They were fed with basal diet (CON), basal diet + 25 mg/kg bacitracin zinc + 5 mg/kg colistin sulfate (AB), 5 g/kg reduction in soybean meal of basal diet + 5 g/kg RBC (RBC1), or 10 g/kg reduction in soybean meal of basal diet + 10 g/kg RBC (RBC2). The results showed that dietary RBC1 improved the body gain/feed intake (G/F) of weaned piglets than the CON diet, and the RBC2 diet improved the average daily gain and G/F than CON and AB diets from days 15 to 28 (*P* < 0.05). Supplementation of RBC2 improved the apparent total tract digestibility of dry matter, nitrogen, and gross energy in weaned piglets compared to controls from days 15 to 28 (*P* < 0.05). Dietary AB, RBC1, and RBC2 enhanced the ileal villus height (VH) and VH/crypt depth (CD), and these two indicators were greater in the RBC2-treated piglets than in the AB- and RBC1-treated piglets (*P* < 0.05). The activity of serum superoxide dismutase (SOD) was enhanced by dietary AB, RBC1, and RBC2 (*P* < 0.05). Serum glutathione (GSH) concentration was elevated by dietary RBC1 and RBC2 (*P* < 0.05). According to 16S rRNA sequence analysis, AB- and RBC2-treated piglets had a higher relative abundance of *Firmicutes* and *Lachnospiraceae* in the colon digesta, and more abundant *Lactobacillus* was found in RBC1-treated piglets, as compared to the CON group. Additionally, RBC2 supplementation increased the α diversity [Chao1, PD-whole-tree, and observed operational taxonomic units (OTUs)] compared to the CON group. Taken together, the dietary RBC improved the growth performance of weaned piglets. In addition, 10 g/kg of RBC2 in the diet achieved better effects on higher ADG, ileal villi morphology, and stronger antioxidant capacity than dietary AB and RBC1 in weaning piglets.

## Introduction

From the onset of weaning, piglets face many stressors including changed diet (from breast nursing to pelleted feed), separation from sows, and living with unacquainted piglets (Montagne et al., 2004; Campbell et al., 2013). Upon weaning, changes in diet have a profound influence on newly weaned piglets since they are equipped with an immature digestive system that cannot digest and absorb feed adequately, leading to the proliferation of *Escherichia coli* easily in the gut (Upadhaya et al., 2018; Shuai et al., 2019). Meanwhile, adverse effects of weaning are also followed by villous atrophy, crypt hyperplasia, and descendent brush border enzyme activity (Pluske et al., 1997). These issues affect the normal growth speed of piglets.

Reportedly, the presence of antibiotics (AB) alleviates diarrhea by killing or preventing the proliferation of pathogenic bacteria (Neuman et al., 2018), prompting animal growth from weaning stress (Ma et al., 2021). In 2006, the European Union banned AB, and China followed suit in 2020 because AB residues in meat products affect the food chain and its resistance genes can be altered by microbiota in the gut and soil (Lin et al., 2017; Ma et al., 2021). Therefore, healthy alternatives are urgently needed to replace AB.

As a fermented product, yeast culture (YC) fermented by live yeast, like *Saccharomyces cerevisiae* (Saied et al., 2011; Dávila-Ramírez et al., 2020), is commonly used as a feed additive, and it contains yeast cell wall polysaccharides, vitamins, minerals, proteins, and enzymes (Song et al., 2021). One of them, marine *Rhodotorula benthica* culture (RBC) is obtained by fermentation of *Rhodotorula benthica* and contains astaxanthin compared to YC but no live *Rhodotorula benthica*. Probiotic *Rhodotorula benthica* unusually secretes astaxanthin which is the source of vitamin A in animals and effectively scavenges free radicals more than vitamin E in the body (Elwan et al., 2018; Wang et al., 2018). According to research, YC has advantages on livestock embodying improved feed intake, average daily gain (ADG), feed conversion ratio (G: F) of finishing pigs, and ADG of fattening lambs (Haddad and Goussous, 2005; Lei and Kim, 2014; Dávila-Ramírez et al., 2020; Song et al., 2021). These benefits from increased nutrient digestibility by YC and mannan-oligosaccharide (yeast component) enhance the intestinal morphology of the small intestine by inducing more goblet cells and higher ileal villous height (De Los Santos et al., 2007; Ayiku et al., 2020). Since *Rhodotorula benthica* is frequently used in the feed of aquatic animals, few studies have been conducted on livestock (Wang et al., 2015). In 2020, a study reported that dietary fermentation product of *Rhodotorula* improved the egg quality and modulated the intestinal microbiota of hens (Sun et al., 2020). After that, Ge et al. (2021) found that mice drinking water with *Rhodotorula mucilaginosa* for half-month exhibited higher immunoglobulin G (IgG) and immunoglobulin A (IgA) in the serum, and a high abundance of *Firmicutes* and *Lactobacillus* in the feces. However, there has been no reported data regarding the effect of RBC on the weaned piglets. Therefore, this trial aimed to explore the effects of RBC supplementation in diets on the growth performance, nutrient digestibility, and intestinal health of weaned piglets.

## Materials and methods

This experiment was conducted at the experiment base in Ya'an (Sichuan, China). All experimental and animal management procedures conformed to the Animal Care and Use Committee of Sichuan Agricultural University (Sichuan, China) and followed animal protection law (approval number: 20160125).

### RBC preparation

Marine *Rhodotorula benthica strains* are detached, selected, and purified after culturing on a slant medium containing seawater. The strains undergo liquid fermentation expansion and transformation for 36 h on a solid fermentation medium under anaerobic conditions. With a temperature of 50–60°C, marine *Rhododendron benthica* break their wall after fermentation for 6–10 h and then are dried under 50–60°C. The product is in powder form and not in pellet form, and its ingredients have been assayed using the Food Standards of China (GB/T 15673-2009 for crude protein, GB/T 23745-2009 for astaxanthin, DB22/T 2462-2016 for methionine, and GB/T 18868-2002 for lysine). The RBC contained crude protein ≥45% (w/w), yeast cell wall polysaccharides (β- glucan and mannan oligosaccharide) ≥100 mg/kg, small peptide ≥5% (w/w), lysine ≥2.8% (w/w), astaxanthin ≥0.01% (w/w), and methionine ≥0.65% (w/w). The product was provided by Prosyn Biological Technology Feed CO., LTD. (Guangzhou, China).

### Experimental design and animal care

The experiment consisted of 96 Duroc × Landrace × Yorkshire weaning piglets (21-day-old) that were split based on body weight and sex into 4 treatment groups. Each treatment group included 6 replicate pens of 4 pigs per pen (2 males: 2 females) with a similar piglet weight of 7.7 ± 0.83 kg. Pigs were supplemented with one of four diets, namely basal diet without any additives (CON), basal diet + 25 mg/kg bacitracin zinc + 5 mg/kg colistin sulfate (antibiotics group, AB), and 5 g/kg reduction in soybean meal of basal diet + 5 g/kg RBC (RBC1 group) or 10 g/kg reduction in soybean meal of basal diet +10 g/kg RBC (RBC2 group). Since the RBC product had more than 45% protein content, we replaced the soybean meal with the same percentage of RBC in the diet. The basal diets [phase 1 (days 1–14) and phase 2 (days 15–28)], shown in Table 1, were formulated to meet the recommendations of the National Research Council (NRC, 2012; USA) for piglets weighing 11 to 25 kg. The period of the feeding trial was 28 days. Pigs were reared in the house where chamber temperature (24–28°C) and humidity of 55-70% were controlled. Additionally, they can drink and consume feed *ad libitum via* a nipple drinker and feeder. Bacitracin zinc (purity of 15% w/w) was purchased from Shenzhen Tongde Veterinary Medicine Co., Ltd (Shenzhen, China), and colistin sulfate (purity of 10% w/w, effectiveness ≥19,000 IU/mg) was bought from Hebei Baipin Biological Technology Co., Ltd. (Heibei, China).

**Table 1:** Ingredients and composition of basal diet.

**Items (as-fed, %)**	**Day 1–14**				**Day 15–28**			
	**CON**	**AB**	**RBC1**	**RBC2**	**CON**	**AB**	**RBC1**	**RBC2**
Corn	18	18	18	18	38.16	38.16	38.16	38.16
Extruded corn	17.99	17.99	17.99	17.99	10	10	10	10
Soybean meal	10.40	10.40	9.90	9.40	18.04	18.04	17.54	17.04
Extruded soybean	13	13	13	13	10	10	10	10
Extruded wheat	5	5	5	5	5	5	5	5
Extruded rice	10	10	10	10	3	3	3	3
Fish meal	5	5	5	5	4	4	4	4
SDPP	3	3	3	3	–	–	–	–
Whey powder	13	13	13	13	7	7	7	7
Soy oil	1.86	1.86	1.86	1.86	2.07	2.07	2.07	2.07
CaHPO_3_	0.45	0.45	0.45	0.45	0.54	0.54	0.54	0.54
Limestone	0.95	0.95	0.95	0.95	0.83	0.83	0.83	0.83
Bacitracin Zinc	0	0.0025	0	0	0	0.0025	0	0
Colistin sulfate	0	0.0005	0	0	0	0.0005	0	0
RBC	0	0	0.5	1	0	0	0.5	1
Salt	0.3	0.3	0.3	0.3	0.3	0.3	0.3	0.3
L-lysine HCl	0.35	0.35	0.35	0.35	0.36	0.36	0.36	0.36
DL-Methionine	0.1	0.1	0.1	0.1	0.07	0.07	0.07	0.07
L-Threonine	0.1	0.1	0.1	0.1	0.11	0.11	0.11	0.11
L-Tryptophan	–	–	–	–	0.02	0.02	0.02	0.02
Vitamin/trace element Premix^a^	0.5	0.5	0.5	0.5	–	–	–	–
Vitamin/trace element Premix^b^	–	–	–	–	0.5	0.5	0.5	0.5
Calculated nutrient composition, %								
NE, kcal/kg	2,596	2,596	2,594	2,591	2,570	2,570	2,567	2,565
CP	20.03	20.03	20.05	20.07	19.78	19.78	19.80	19.82
Ca	0.7	0.7	0.7	0.7	0.7	0.7	0.7	0.7
Available phosphorus	0.4	0.4	0.4	0.4	0.4	0.4	0.4	0.4
Calculated standardized ideal digestible value, %								
d-Lys	1.20	1.20	1.20	1.20	1.18	1.18	1.18	1.18
d-Met	0.39	3.9	3.9	3.9	3.6	3.6	3.6	3.6
d-Thr	0.79	0.79	0.79	0.79	0.73	0.73	0.73	0.73
d-Trp	0.23	0.23	0.23	0.23	0.2	0.2	0.2	0.2
Analyzed nutrient values, %								
DE, MJ/kg	14.91	14.91	14.94	14.96	14.73	14.72	14.80	14.81
CP	20.11	20.10	20.13	20.15	19.82	19.82	19.88	19.87
Lys	1.32	1.32	1.35	1.37	1.30	1.30	1.34	1.35
Met+Cys	0.59	0.59	0.61	0.62	0.61	0.60	0.61	0.63

a Provided per kg of complete diet: vitamin A, 2,200 IU; vitamin D3, 220 IU; vitamin E, 16 IU; vitamin K3, 0.5 mg; vitamin B12, 0.0175 mg; riboflavin, 3.5 mg; niacin, 30 mg; thiamine, 10 mg; choline, 500 mg; folic acid, 0.3 mg; vitamin B1, 1 mg; vitamin B6, 7 mg; biotin, 0.05 mg; Zn (as ZnSO_4_), 100 mg; Mn (as MnO_2_), 4 mg; Fe (as FeSO_4_·7H_2_O), 100 mg; Cu (as CuSO_4_·5H_2_O), 6 mg; I (as KI), 0.14 mg; Se (as Na_2_SeO_3_·5H_2_O), 0.3 mg.

b Provided per kg of complete diet: vitamin A, 1,750 IU; vitamin D3, 220 IU; vitamin E, 11 IU; vitamin K3, 0.5 mg; riboflavin, 3 mg; niacin, 30 mg; thiamine, 1 mg; d-pantothenic, 9 mg; choline, 400 mg; vitamin B_12_, 15 μg; folic acid, 0.3 mg; vitamin B6, 3 mg; biotin, 0.05 mg; Zn (as ZnSO_4_), 80 mg; Mn (as MnO_2_), 3 mg; Fe (as FeSO_4_·7H_2_O), 100 mg; Cu (as CuSO_4_·5H_2_O), 5 mg; I (as KI), 0.14 mg; Se (as Na_2_SeO_3_·5H_2_O), 0.25 mg.

### Growth performance and diarrhea score

Before checking individual body weight (BW) on the morning of days 15 and 29, the feed was withdrawn 8 h in advance. The given feed and the residual feed by individual pen were recorded daily. Growth performance parameters included ADG, average daily feed intake (ADFI), and G/F which was calculated by dividing the ADG by ADFI. The dead piglets were recorded daily to revise performance indices.

Diarrhea scores for each piglet were checked twice per day at 09:00 h and 17:00 h, respectively. The calculation for diarrhea rate was done using the formula of Giang et al. (2012). Feces consistency scores criterion was as follows: 0, hard bar or granular; 1, soft stools but shapeable; 2, unshaped; 3, watery stool. According to the criterion, the piglet was in diarrhea when the diarrhea score was not <2 scores. The diarrhea rate = total number of piglets with diarrhea/ (number of all piglets × days of this experiment) × 100%.

### Sample collection

Immediately after BW measurement on days 15 and 29, one female piglet/pen with BW closest to the average BW of treatment was selected. Blood samples taken from the anterior vena cava were put in two 5 mL vacuum tubes (with /without heparin sodium). Then, all heparin sodium tubes were immediately sent to Sichuan Agricultural University Pet Hospital (Ya'an, China) to determine the number of white blood cells (WBCs) and lymphocytes (LYMs) through a BC-2600 automatic blood cell analyzer (Mindray, China) for quantitative analysis. The unit of WBC was 10^9^/L, and the LYM was %. The remaining tubes without heparin sodium were centrifuged (3,500 × *g*, 4°C for 15 min) to obtain serum that was analyzed for immunoglobulin and antioxidant indicators and stored (−20°C).

The digestion trial was implemented twice from days 8 to 14 and from days 22 to 28. Chromic oxide (Cr_2_O_3_) as an analytical marker, was mixed in diets (0.25% w/w). After 4-day adaptation, fresh fecal samples were obtained by stimulating the anal sphincters from 2 piglets in each pen from days 12 to 14 and from days 26 to 28; then 3-day feces per pen were mixed in equal proportions and stored (−20°C).

At the termination of this trial, 7 piglets from each treatment with BW closest to the average BW of treatment were chosen and euthanized by injecting sodium pentobarbital according to the manual. Their intestine was stripped from the mesentery and immediately placed on ice. Sections (about 2 cm) from the middle of the individual duodenum, jejunum, and ileum were cut and placed into 4% (v/v) paraformaldehyde solution for histomorphometry measurement. Colonic chyme was aseptically collected into sterile Eppendorf tubes and stored in liquid nitrogen, then removed to the fridge at −80°C for analyzing microbiota.

### Serum IgG, IgM, and antioxidant index determination

Concentrations of serum IgG and IgM were determined with porcine IgG and IgM ELISA kits (Nanjing Jiancheng Bioengineering Institute, Nanjing, China) with a microplate reader (SpectraMax^®^190, Molecular Devices, USA) at 450 nm. The logistic curves of IgG and IgM were built according to the manufacturer's instructions, and used the analysis software of ELISA calc. The limits for IgG and IgM concentrations were 0.3–90 mg/mL and 0.1–30 mg/mL, respectively. Coefficients of intra- and inter-sample variations were all <10 and 12% for IgG and IgM, respectively.

The content of reduced glutathione (GSH) and malondialdehyde (MDA) and the activity of superoxide dismutase (SOD) were assayed using specific assay kits (Product code: A006-2, A003-1, A001-3, Nanjing Institute of Jiancheng Biological Engineering, Nanjing, China) according to the manufacturer's instruction. A microplate reader (SpectraMax^®^190, Molecular Devices, USA) with the absorbance of 405, 532, and 450 nm for GSH, MDA, and SOD, respectively was recommended to read the numbers. Parallel determination was conducted for each sample.

### Detection of intestinal morphology

The fixed jejunal segments were rinsed with running water for 30 min and subsequently dehydrated with absolute ethanol at varying concentrations. These tissues were cleared with xylene, embedded in wax, and sliced into 5 μm-thick slices using a Leica RM2235 microtome (Leica, Germany). Finally, these slices were dewaxed and stained with hematoxylin-eosin. For each well-oriented villus, 10 measurements were recorded for both villus height (VH) and crypt depth (CD) using Image Pro Plus 6.0. The average of these 10 measurements was taken to represent the VH and CD for each tissue. The V/C ratio was obtained by dividing the VH by the CD value.

### 16S ribosomal RNA (rRNA) sequencing

The frozen colonic digesta at −80°C were thawed and extracted for total DNA using QIAamp PowerFecal Pro DNA kit (Qiagen, Hilden, Germany) following the manufacturer's instructions. The DNA samples for purity and integrity examination were analyzed using a NanoDrop 2000 spectrophotometer (Thermo, Waltham, USA) and electrophoresis (2% w/v gel). DNA concentration was quantified by Equalbit1 × dsDNA HS Assay Kit (Vazyme Biotech Co., Ltd., Nanjing, China) and diluted to 1 ng/μL, and purified by Qiagen Gel extraction kit (Qiagen, Germany). Library construction using Ion Plus Fragment Library Kit 48 rxns (Thermos Scientific, USA) and Illumina MiSeq sequencing on IonS5™ was performed in Novogene Bioinformatics Technology (Beijing, China). The hypervariable region primers of V3-V4 and amplicons library of 16S rRNA kept consistent with our previous description (Zhang Q. et al., 2020). Raw reads were obtained from the spliced sequences by removing the primer sequences and barcode, and primers and adapters were removed from the raw reads using the Cutadapt (V1.9.1), then clean reads were obtained. Operational taxonomic units (OTUs) were clustered by clean reads using Uparse software (http://www.drive5.com/uparse/) with 97% identity. The feature classifiers were trained according to SILVA 132 database (http://www.arb-silva.de/). Alpha and beta diversities were analyzed using QIIME (version 1.9.1; Caporaso et al., 2010). Differential taxonomic markers for each group were determined using linear discriminant analysis effect size (LEfSe; Segata et al., 2011).

### Statistical analysis

The data were analyzed by mixed procedure with PDIFF (SAS 9.4 Inst., Inc., Cary, NC). The results were expressed as means ± pooled standard error. The *P-*value was used for the comparison of every 2 treatment groups from the least square means. *P* < 0.05 was considered significant. Replicate pen was the statistics for growth performance, diarrhea, and ATTD. Intestinal morphology, serum indices, and grouped microbial were analyzed using each euthanized piglet as an experiment unit. The relative abundance of microorganisms was used as the result of Metastat analysis. The Principal Co-ordinates Analysis (PCoA) was visualized by the “vegan” package of R (2.15.3).

## Results

### Growth performance and diarrhea score

Supplementary RBC1 in the diet notably improved (*P* < 0.05) the G/F compared with the CON diet (Table 2) from days 15 to 28 and from days 1 to 28. Higher ADG and G/F (*P* < 0.05) were found in the RBC2-treated piglets than in the controls from days 15 to 28 and from days 1 to 28 (*P* < 0.05). Compared with AB-treated piglets, the piglets fed with RBC2 diet had higher ADG and G/F from days 15 to 28 and greater G/F from days 1 to 28 (*P* < 0.05). However, the differences were not observed between CON vs. AB, AB vs. RBC1, or RBC1 vs. RBC2 groups. As shown in Table 3, diarrhea scores of weaning piglets were similar among groups.

**Table 2:** Effect of dietary RBC and AB supplementation on the growth performance of weaning piglets.

**Items**	**CON**	**AB**	**RBC1**	**RBC2**	**SEM^a^**	***P*** **-value**					
						**CON vs. AB**	**CON vs. RBC1**	**CON vs. RBC2**	**AB vs. RBC1**	**AB vs. RBC2**	**RBC1 vs. RBC2**
BW, kg											
Day 0	7.75	7.69	7.69	7.70	0.37	0.910	0.915	0.930	0.995	0.980	0.985
Day 14	10.41	10.76	10.60	10.72	0.46	0.595	0.775	0.639	0.804	0.950	0.853
Day 28	16.83	17.40	17.78	18.30	0.63	0.534	0.304	0.117	0.678	0.326	0.565
Day1–14											
ADG, g/d	190	220	208	216	21	0.327	0.556	0.394	0.688	0.894	0.788
ADFI, g/d	265	292	290	295	24	0.447	0.476	0.393	0.962	0.923	0.886
G/F	0.728	0.741	0.723	0.734	0.040	0.814	0.928	0.914	0.745	0.898	0.843
Day 15–28											
ADG, g/d	458	474	513	541	23	0.638	0.108	0.018	0.243	0.049	0.381
ADFI, g/d	721	747	714	727	50	0.709	0.937	0.931	0.652	0.774	0.869
G/F	0.638	0.649	0.724	0.750	0.028	0.772	0.039	0.010	0.070	0.019	0.525
Day 1–28											
ADG, g/d	324	347	360	379	17	0.362	0.152	0.035	0.583	0.200	0.452
ADFI, g/d	493	519	502	511	32	0.559	0.832	0.691	0.709	0.850	0.853
G/F	0.661	0.674	0.724	0.743	0.019	0.623	0.032	0.007	0.086	0.020	0.481

a Pooled standard error of means, n = 6.

**Table 3:** Effect of dietary RBC and AB supplementation on the diarrhea score of weaning piglets (%).

**Items**	**CON**	**AB**	**RBC1**	**RBC2**	**SEM^a^**	***P*** **-value**					
						**CON vs. AB**	**CON vs. RBC1**	**CON vs. RBC2**	**AB vs. RBC1**	**AB vs. RBC2**	**RBC1 vs. RBC2**
Day 0–14	0.222	0.344	0.249	0.189	0.155	0.584	0.903	0.881	0.670	0.487	0.786
Day 15–28	0.753	0.523	0.524	0.429	0.135	0.243	0.243	0.104	0.999	0.624	0.624
Day 0–28	0.488	0.434	0.386	0.309	0.113	0.739	0.532	0.276	0.769	0.443	0.634

a Pooled standard error of means, n = 6.

### Apparent total tract digestibility

Compared with the CON group, the piglets in the AB group had higher ATTD of DM (*P* < 0.05) from days 1 to 14 and from days 15 to 28 (Table 4), and this index was also higher in the RBC1 group from days 15 to 28 (*P* = 0.004). The ATTD of GE from days 1 to14, DM, N, and GE from days 15 to 28 were higher (*P* < 0.05) in the RBC2 group than those in the CON group.

**Table 4:** Effect of dietary RBC and AB supplementation on nutrients digestibility of weaning piglets (%).

**Items**	**CON**	**AB**	**RBC1**	**RBC2**	**SEM^a^**	***P*** **-value**					
						**CON vs. AB**	**CON vs. RBC1**	**CON vs. RBC2**	**AB vs. RBC1**	**AB vs. RBC2**	**RBC1 vs. RBC2**
Day 1–14											
DM	81.40	83.18	82.28	82.45	0.60	0.047	0.307	0.226	0.300	0.398	0.843
N	81.79	83.05	83.37	83.18	0.76	0.255	0.158	0.210	0.771	0.905	0.864
GE	79.80	81.80	81.06	83.29	0.98	0.162	0.373	0.020	0.596	0.295	0.122
Day 15–28											
DM	77.36	80.67	81.50	82.22	0.90	0.017	0.004	0.001	0.526	0.237	0.573
N	78.19	79.39	80.27	82.36	1.12	0.458	0.206	0.016	0.587	0.076	0.202
GE	77.51	78.50	80.38	81.90	1.17	0.559	0.101	0.016	0.274	0.055	0.370

a Pooled standard error of means, n = 6.

### Intestinal morphology

Compared to piglets in the CON group, the basal diet supplemented with AB or RBC1 was conducive to higher VH and greater V/C ratio of piglets in the ileum (*P* < 0.01, Table 5); while dietary RBC2 improved the jejunal VH, ileal VH, and V/C (*P* < 0.05). Additionally, AB-treated piglets exhibited a higher (*P* < 0.05) duodenal VH and V/C ratio, as compared to RBC1-treated piglets. However, a greater ileal VH and V/C ratio of piglets were found in the RBC2 group, as compared to the AB group (*P* < 0.001). The RBC2 dose exhibited a better effect than the RBC1 dose on piglets, showing a higher VH in the duodenum and ileum, shallower ileal CD, and greater ileal V/C ratio (*P* < 0.05).

**Table 5:** Effect of dietary RBC and AB supplementation on the intestinal morphology of weaning piglets.

**Items**	**CON**	**AB**	**RBC1**	**RBC2**	**SEM^a^**	***P*** **-value**					
						**CON vs. AB**	**CON vs. RBC1**	**CON vs. RBC2**	**AB vs. RBC1**	**AB vs. RBC2**	**RBC1 vs. RBC2**
Duodenum											
VH, μm	373.17	381.00	367.17	378.83	4.47	0.229	0.353	0.380	0.041	0.735	0.078
CD, μm	242.00	236.50	243.33	239.33	3.41	0.267	0.785	0.586	0.172	0.563	0.416
V/C ratio	1.54	1.61	1.51	1.58	0.02	0.051	0.369	0.252	0.007	0.382	0.049
Jejunum											
VH, μm	320.17	330.33	320.83	327.67	5.63	0.216	0.934	0.358	0.247	0.741	0.401
CD, μm	172.67	171.17	166.17	163.67	3.43	0.721	0.196	0.079	0.316	0.138	0.613
V/C ratio	1.86	1.93	1.93	2.01	0.05	0.263	0.261	0.029	0.996	0.243	0.245
Ileum											
VH, μm	279.33	318.83	321.67	351.00	3.58	<0.001	<0.001	<0.001	0.582	<0.001	<0.001
CD, μm	153.00	149.67	156.33	146.83	2.84	0.417	0.417	0.141	0.113	0.489	0.028
V/C ratio	1.83	2.14	2.06	2.42	0.05	0.013	<0.001	0.002	0.246	<0.001	<0.001

a Pooled standard error of means, n = 7.

### Blood characteristics and antioxidant properties

Serum IgG, IgM, WBC, and LYM were not different among groups on days 14 and 28 (Table 6). Serum SOD activity on day 14 from piglets fed with the AB diet was elevated (*P* = 0.001), as shown in Table 7 when compared with the controls. The RBC1-treated piglets had higher concentration of (*P* < 0.01) serum GSH on days 14 and 28 and the SOD activity on day 28, as compared to the piglets in the CON group, while piglets fed with the RBC2 diet exhibited a higher GSH concentration on days 14 and 28 and the SOD activity on day 14 compared with the controls (*P* < 0.001). The GSH concentration on days 14 and 28 was increased (*P* < 0.001), while the SOD activity was reduced on day 14 but elevated on day 28 in piglets fed with RBC1 diet (*P* < 0.05), as compared to that in the AB group; additionally, RBC2-treated piglets had a higher GSH concentration on days 14 and 28 than AB-treated piglets (*P* < 0.001). Moreover, when compared to RBC1-treated piglets, the dietary RBC2 induced higher SOD activity on days 14 and 28 and GSH activity on day 14 (*P* < 0.05).

**Table 6:** Effect of RBC and AB supplementation on the blood profiles of weaning piglets.

**Items**	**CON**	**AB**	**RBC1**	**RBC2**	**SEM^a^**	***P*** **-value**					
						**CON vs. AB**	**CON vs. RBC1**	**CON vs. RBC2**	**AB vs. RBC1**	**AB vs. RBC2**	**RBC1 vs. RBC2**
Day 14											
IgG, μg/L	2.62	2.85	2.80	3.24	0.23	0.475	0.581	0.069	0.869	0.249	0.191
IgM, μg/L	0.198	0.298	0.272	0.338	0.07	0.301	0.446	0.154	0.780	0.676	0.488
WBC, 10^9^ /L	23.02	21.67	22.48	21.56	0.74	0.210	0.612	0.178	0.444	0.921	0.389
LYM, %	73.82	74.28	74.18	76.15	2.09	0.876	0.903	0.439	0.973	0.535	0.513
Day 28											
IgG, μg/L	4.20	4.23	4.67	4.40	0.42	0.960	0.435	0.687	0.464	0.720	0.702
IgM, μg/L	0.22	0.25	0.28	0.37	0.02	0.380	0.114	0.172	0.459	0.609	0.816
WBC, 10^9^ /L	23.85	24.25	23.17	22.47	1.94	0.886	0.808	0.621	0.699	0.525	0.801
LYM, %	15.22	15.95	15.07	15.69	1.66	0.757	0.949	0.843	0.709	0.911	0.793

a Pooled standard error of means, n = 6.

**Table 7:** Effect of dietary RBC and AB supplementation on serum antioxidant properties of weaning piglets.

**Items**	**CON**	**AB**	**RBC1**	**RBC2**	**SEM^a^**	***P*** **-value**					
						**CON vs. AB**	**CON vs. RBC1**	**CON vs. RBC2**	**AB vs. RBC1**	**AB vs. RBC2**	**RBC1 vs. RBC2**
Day 14											
GSH, ug/mL	613.11	598.41	653.35	711.85	6.48	0.124	< 0.001	< 0.001	< 0.001	< 0.001	< 0.001
MDA, nmol/mL	6.78	6.25	6.26	6.35	0.31	0.239	0.247	0.332	0.985	0.829	0.844
SOD, U/mL	98.77	108.79	101.84	110.21	1.78	0.001	0.236	< 0.001	0.012	0.577	0.003
Day 28											
GSH, ug/mL	595.50	602.19	673.85	663.03	3.67	0.158	< 0.001	< 0.001	< 0.001	< 0.001	0.051
MDA, nmol/mL	6.55	6.05	6.41	6.46	0.17	0.055	0.570	0.735	0.159	0.105	0.818
SOD, U/mL	104.82	106.55	113.70	106.40	1.55	0.440	0.001	0.480	0.004	0.946	0.003

a Pooled standard error of means, n = 6.

### Microbial composition in colonic digesta

**Figure 1:**
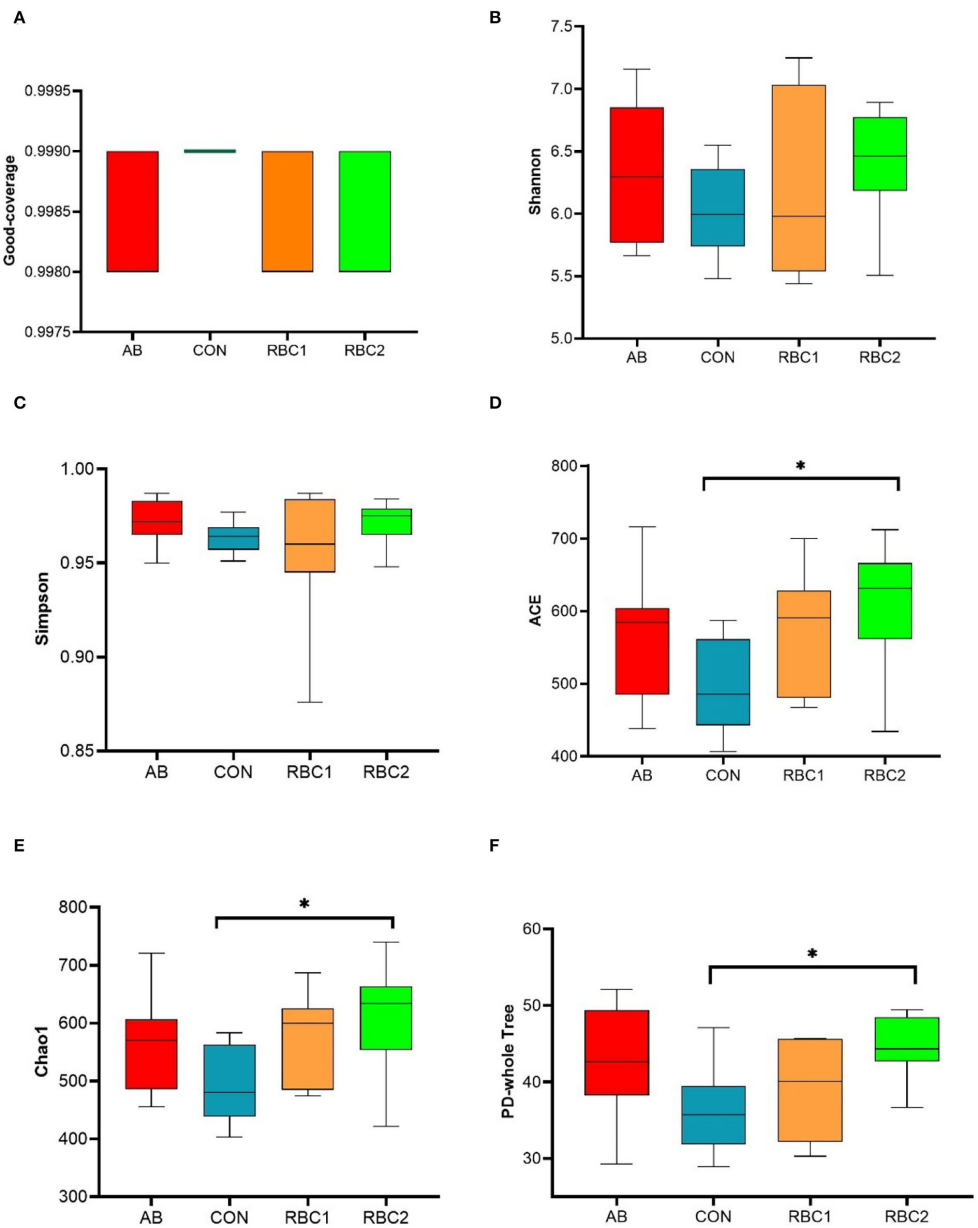
Effect of dietary RBC and antibiotics (AB) supplementation on the α and β diversity in the colonic digesta of weaning piglets. **(A–F)** Goods-coverage, Shannon, Simpson, ACE, Chao1, and Ph-whole tree indices of α diversity. *n* = 7 for each treatment. CON, basal diet; AB, basal diet + 25 mg/kg bacitracin zinc+5 mg/kg colistin sulfate; RBC1, 5 g/kg reduction in soybean meal of basal diet +5 g/kg RBC; RBC2, 10 g/kg reduction in soybean meal of basal diet +10 g/kg RBC. *Means *P* < 0.05.

**Figure 2:**
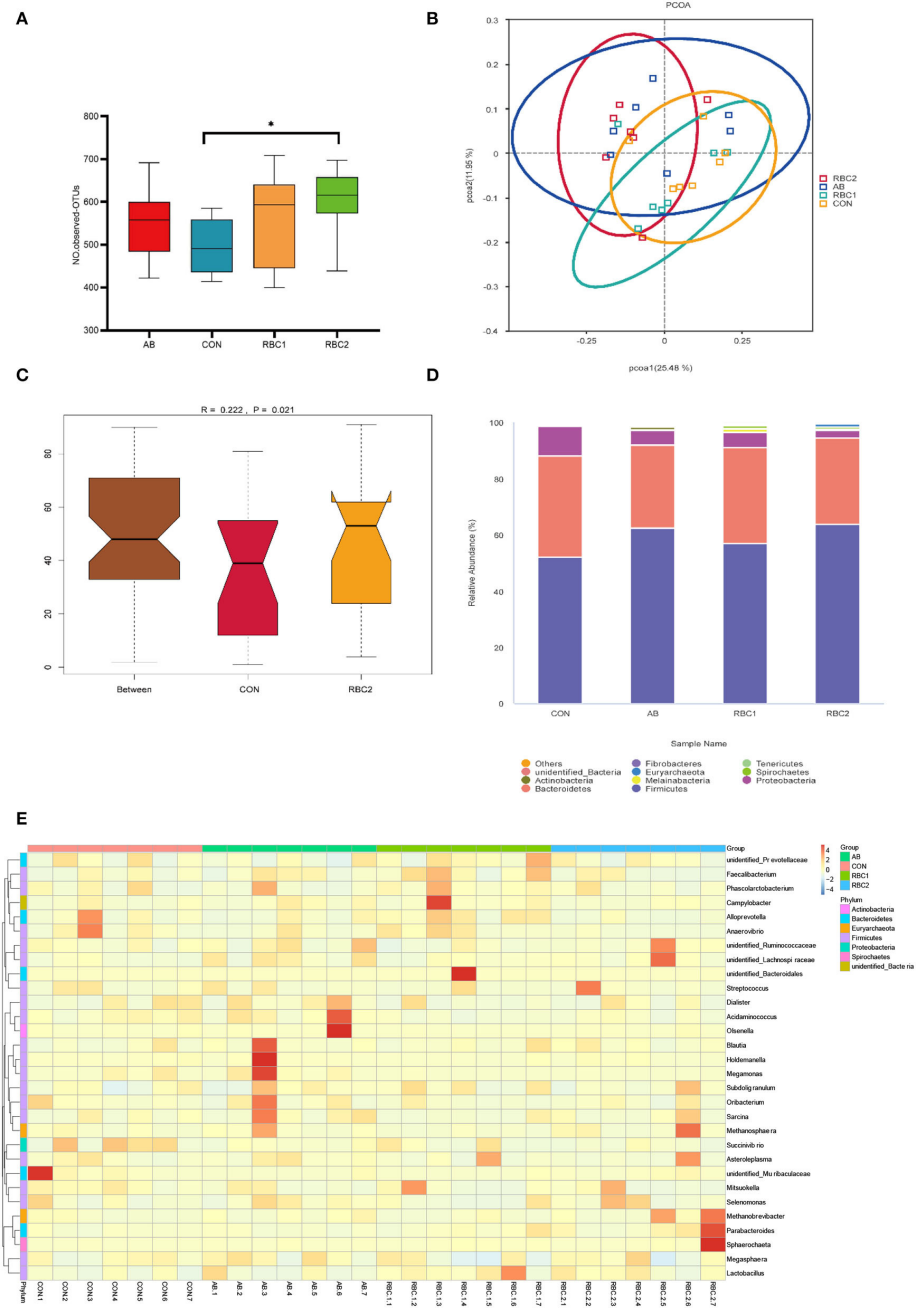
Effect of dietary AB and RBC on the relative abundance of microbiota at the phylum and genus level, and PCoA and Adonism of weaned piglets. **(A)** Operational taxonomic units (OTUs) number in each treatment group. **(B)** Principal coordinates analysis (PCoA). **(C)** Analysis of similarities (ANOSIM) between CON and RBC2 group. **(D)** Microbiota abundance at the phyla level. **(E)** Microbiota abundance at the genus level. *n* = 7 for each treatment. CON, basal diet; AB, basal diet + 25 mg/kg bacitracin zinc+5 mg/kg colistin sulfate; RBC1, 5 g/kg reduction in soybean meal of basal diet + 5 g/kg RBC; RBC2, 10 g/kg reduction in soybean meal of basal diet + 10 g/kg RBC. *Means *P* < 0.05.

In the present study, an average of 77,665 raw reads and 72,560 clean reads were obtained for each group with the length of the sequences ranging between 409 and 420 bp. The sequence number at the OTU level was 440,028 (Supplementary Figure S1), and it tended to reach a plateau, suggesting that the majority of OTU samples were captured. The α diversity indices in Figures 1A–F, 2A are represented by richness (observed-OTUs, ACE, and Chao1), diversity (Simpson and Shannon), goods-coverage (sequencing depth index), and PD_ whole_ tree (index of phylogenetic diversity). A sharp contrast between PRC2 and CON groups showed an increase in Chao1, PD-whole-tree, and observed-OTUs (*P* < 0.05) in the RBC2 group. Shannon and Simpson indicators were similar among groups (*P* > 0.05). The unweighted Unifrac PCoA plot (Figure 2B) visually indicated no separation of microbial communities among groups (*P* > 0. 05). The ANOSIM R-value of unweighted Unifrac distance (*R* = 0.026, *P* = 0.027; Figure 2C) was close to zero, which represented the weak separation of the microbiota community between CON and RBC2 groups.

### Relative abundance of microbiota at phylum and genus levels

The top 10 predominant microorganisms at the phylum level depicted in Figure 2D (Supplementary Table S1) showed that bacteria belonging to *Firmicutes* (51.99%), *Bacteroidetes* (31.04%), and *Proteobacteria* (5.27%) occupied more than 88% of the total sequences. Supplementation of RBC and AB seemed to change the microbiota abundance as *Firmicutes* (44.48%) *and Proteobacteria* (9.95%) in the CON group, *Firmicutes* (55.28%) *and Proteobacteria* (3.97%) in the AB group, and *Firmicutes* (50.86%) *and Proteobacteria* (4.95%) in the RBC1 group, *Firmicutes* (57.32%) *and Proteobacteria* (2.18%) in the RBC2 group. The relative abundance of *Firmicutes* in the AB and RBC2 groups was higher (*P* = 0.015) when compared with the CON group. The relative abundance of *Euryarchaeota* was higher (*P* < 0.05) in the RBC2 group than in the RBC1 and CON groups, and in the AB group than in the RBC1 group, while that of *Proteobacteria* was lower in the RBC2 group than in the CON group (*P* = 0.019).

At the genus level (Figure 2E, Supplementary Table S2), a heatmap showed the top 30 microorganisms in the genus level among the total number of 185 genera. *Lactobacillus* (6.73%), *Prevotella_9* (9.69%), *Mitsuokella* (4.86%), *Succinivibrio* (4.65%), and *Dialister* (3.94%) had high relative abundance in all colon digesta samples. Supplementation of RBC1 and RBC2 diets both lowered the relative abundance of *Acidaminococcus*, as compared to the CON and AB diets (*P* < 0.05). The *Succinivibrio* population was lowered in the RBC2 group compared to the CON group (*P* = 0.023). The relative abundance of *Prevotella, Prevotellaceae_UCG-*003, and *Ruminococcus* were enriched in the RBC1-treated piglets than in the piglets of the AB group (*P* < 0.05). *Rikenellaceae_RC9_gut*_group and *g__UCG-*002 population were enriched in the RBC2 group compared with the RBC1group (*P* < 0.05).

### LEfSe analysis in the OUT level

**Figure 3:**
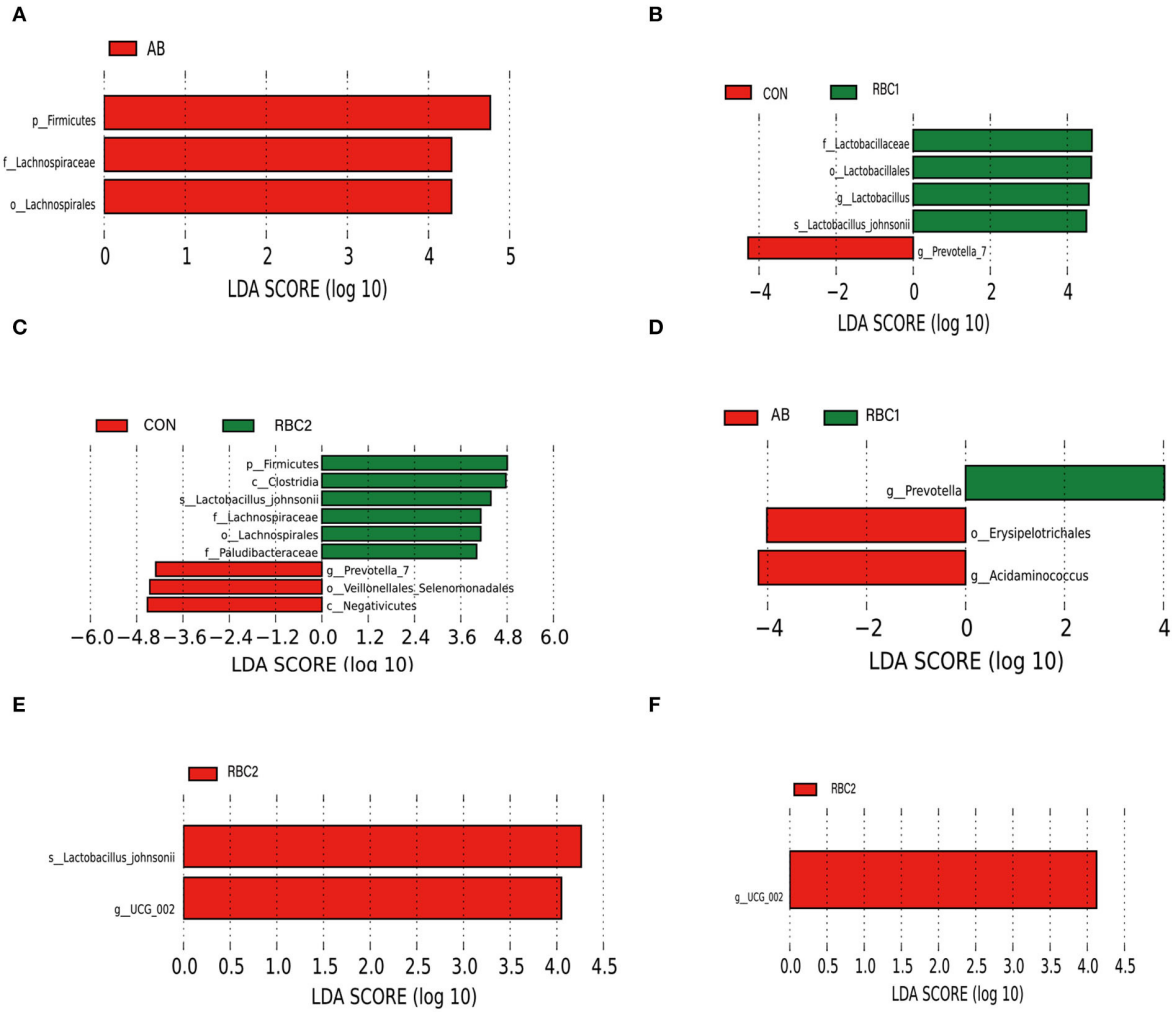
LEfSe analysis of digesta microbiota. **(A)** CON vs. AB group, **(B)** CON vs. RBC1 group, **(C)** CON vs. RBC2 group, **(D)** AB vs. RBC1group, **(E)** AB vs. RBC2 group, **(F)** RBC1 vs. RBC2 group. *n* = 7 for each treatment. CON, basal diet; AB, basal diet + 25 mg/kg bacitracin zinc+5 mg/kg colistin sulfate; RBC1, 5 g/kg reduction in soybean meal of basal diet +5 g/kg RBC; RBC2, 10 g/kg reduction in soybean meal of basal diet +10 g/kg RBC.

LEfSe analysis is used to assess the represented biomarker for comparison among groups. Linear discriminant analysis (score set 4) depicted in Figure 3A shows that the AB group had a higher relative abundance of *p_Firmicutes, f* _*Lachnospiraceae*, and *o*_ *Lachnospirales* in comparison to the CON group. While in Figure 3B, the RBC1 group exhibited enriched *f* _*Lactobacillaceae, o*_*Lactobacillales, g_Lactobacillus*, and *s_Lactobacillus_ johnsonii*, as compared to the CON group whereas g_*Provotella*_7 was abundant. In Figure 3C, *p_Firmicutes, c*_*Clostridia, s*_*Lactobacillus_ johnsonii, f_Lachnospiraceae, o_Lachnospirales*, and *f_Paludibacteraceae* were increased in the RBC2-treated piglets when compared to the CON group where *g*_*Prevotella_*7, *o*_*Veillonellales_ Selenomonadales*, and *c_ Negativicutes* were enriched. Compared to the microbiota abundance of AB-treated piglets (Figure 3D), piglets fed with RBC1 diet had more *g*_*Prevotella* populations in the colon, while *o*_*Erysipelotrichales* and *g*_ *Acidminococcus* were enriched in the AB-treated piglets; meanwhile, *s*_*Lactobacillus_johnsonii* and *g*_*UCG_*002 were enriched in the colon digesta from piglets fed withRBC2 diet (Figure 3E). When compared to RBC1-treated piglets (Figure 3F); more abundant *g*_*UCG_*002 was found in piglets fed with RBC2 diet.

## Discussion

Natural marine red yeast strains are rich in protein, astaxanthin, and enzyme and characterize high salt tolerance; therefore, they are commonly used as feed additives in aquaculture (Matrosova and Politaeva, 2021; Yun et al., 2021). RBC without live yeast also has the above features but it has fewer applications on animals. For a long time, overcoming weaning syndrome in piglets has always been a research hotspot; thus, we look forward to the influences of RBC on weaning piglets.

In this study, RBC1 and RBC2 supplementation as the alternative to soybean meal in the basal diet improved the ADG and G/F of piglets except in the phase of days 1–14. This result was partially in line with the findings of Dávila-Ramírez et al. (2020), that dietary *Saccharomyces cerevisiae* culture at the dose of 0.2% and 0.3% both improved the final BW, ADG, and ADFI of growing pigs, and the results of Lee et al. (2018), showed that 0.5% fermented YC improved the ADG and feed intake of weaned piglets. Moreover, supplementation of RBC at the dose of 10 g/kg improved the ADG and G/F of piglets more than the effect of diet-containing AB. To our knowledge, the feed intake is a vital factor for growth speed (Bruininx et al., 2001), but there was a similar ADFI among groups. Indeed, supplementary RBC and AB in the diet did not change the dietary nutritional value or physical character; consequently, there was no improvement in the feed intake, and it was normal. Meanwhile, the immunity response of piglets was not affected by RBC although RBC could promote serum protein and albumin synthesis (Saied et al., 2011). Of note, Cui et al. (2019) and Song et al. (2021) reported that the elevated growth may not be related to the feed intake but to digestibility.

The nutrient digestibility and absorption and piglet diarrhea indicators are also decisive factors for growth performance (Haddad and Goussous, 2005; Hu et al., 2015). In this study, although the diarrhea scores were not affected by dietary additives, the RBC2 supplementation improved DM, GE, and N digestibility, and greater DM digestibility was also found in AB and RBC1-treated piglets. These agreed with the effects of fermented YC products on elevating the nutritional digestibility and growth performance of lambs without affecting intakes (Lei and Kim, 2014; Zhang et al., 2019). RBC product contains biologically active substances, such as bioactive peptide, which can be absorbed by villous epithelial cells of the small intestine and stimulates the activity of chorionic brush border enzymes (Wang et al., 2017), as well as modulates the nutrient digestibility (Bao and Wu, 2021). Additionally, the protein from fermented RBC is the most water-soluble protein with easy absorption, which may increase the ATTD of N (Hu et al., 2008). Moreover, RBC containing lysing also prompts nutrient absorption (Zeng et al., 2013). Of course, it was no doubt that improvements in nutritional absorption and growth performance were closely related to the improved gut environment induced by ingredients of RBC.

Intestinal morphology indices that are common standards to estimate the ability of the intestine for nutrient digestion and absorption are widely reported. The potential of yeast derivative that induces morphology on higher VH and greater V/C ratio of weaned piglets or growing-finishing pigs (Zhang et al., 2005; Gang et al., 2017; He et al., 2021), and goblet cell density of broilers (Reisinger et al., 2012) have been reported. In the current study, the RBC induced a higher ileal V/H and V/C ratio, and the dietary RBC2 greatly affected the ileal morphology more than the AB and RBC1 diet. Earlier studies prove that mannan-oligosaccharides, a component of yeast cells, prevent the villi from contacting pathogens (Sims et al., 2004; Castillo et al., 2008), like *Clostridium perfringens* and *Salmonella*, is beneficial to maintain the normal function of mucosa and reduce diarrhea during the weaning period (Gao et al., 2008). Admittedly, AB had the capability of improving the VH and V/C in the small intestine (Oliver and Wells, 2013; Long et al., 2018) due to inhibiting harmful bacteria. This result exhibited that the supplementation of RBC at the dose of 10 g/kg improved the ileal VH and V/C compared to AB-treated piglets. On the one hand, a higher villus manifests faster cell renewal and more mature epithelia and increases the absorptive area of the villus (Gao et al., 2008). On the other hand, taller VH improves the secretion of digestive enzyme activity from the tips of the villi (Hampson, 1986), leading to elevated ATTD. Additionally, a higher magnitude of the V/C ratio directly indicates the strength of absorption (Pluske et al., 1996). Hence, in this research, the larger V/C ratio in RBC treatments may contribute to the higher digestibility.

Oxidant stress results in weaker growth performance and lower nutrient digestibility of weaned pigs (Yuan et al., 2007). In the current study, the dietary inclusion of RBC improved the GSH content more than CON and AB diets on days 14 and 28, with RBC2 achieving the optimal effect. The GSH can be an index to evaluate the antioxidant capacity of the body because it possesses vital physiological functions, such as free radical scavenging, detoxication, and maintaining cellular immunity (Tang et al., 2008). Supplementation of AB, RBC1, and RBC2 enhanced the SOD activity in the serum. SOD is a type of antioxidant enzyme that removes harmful free radicals from the cellular environment (Fattman et al., 2003). According to researchers, the astaxanthin that is present in the RBC with the structure of a hydroxyl and a keto group can neutralize free radicals, and scavenge singlet oxygen, peroxyl, and hydroxyl radicals (Hardo et al., 2020), and this function has been proved in finishing pigs and fishes (Lei and Kim, 2014; Li et al., 2017). Subsequently, methionine was reported to eliminate reactive oxygen species by methionine residues or through GSH synthesis (Zeitz et al., 2017). The RBC containing methionine is attributed to its higher GSH; this was not observed in the AB group, which was in response to stronger antioxidant capacities in the RBC groups than in the AB group.

Reportedly, gut microbial populations in pigs are influenced by dietary ingredients, such as trace elements, AB, and beneficial bacteria (Zhang et al., 2016). Meanwhile, they also modulate nutrient digestibility and serve as an important barrier pathogen (Fouhse et al., 2016).

The increased PD_ whole_ tree, observed-OTUs, and Chao1 in the RBC2 group indicated that the dietary RBC at the dose of 10 g/kg increased the richness of colonic flora compared to the controls. At the phylum level, *Firmicutes*, more abundant in AB and RBC2 group, are positively interrelated in energy absorption and produce acetate and lactate (Hu et al., 2020; Oh et al., 2021). Also, *Euryarchaeota* enriched in the RBC2 is well-known for working at metabolizing nutrients and metabolites of other bacteria, resulting in elevated short-chain fatty acid concentration, such as acetate (Primec et al., 2019). The abundant 2 phyla may be connected to improved GE digestibility of RBC piglets. The increased *Acidaminococcus* in the AB group, *Lachnospiraceae*, and *Paludibacteraceae* in the RBC2 group, involve fermenting fibers, polysaccharides, and glutamate (Ren et al., 2019; Tian et al., 2019; Zhang L. et al., 2020; Ryazanov et al., 2021). As we know, RBC contains abundant yeast cell polysaccharides, which may stimulate the proliferation of *Euryarchaeota, Lachnospiraceae*, and *Paludibacteraceae*. Importantly, *Lachnospiraceae* and *Paludibacteraceae* protect intestinal cells from injury (Berger et al., 2021). In addition, the abundant genus, *Lactobacillus* in the RBC1 group is well-known to improve nutrient digestibility and against intestinal *Escherichia coli* in weaned piglets (Dowarah et al., 2017; Wang et al., 2019). Moreover, *Lactobacillus_ johnsonii* belonging to the genus, *Lactobacillus* is useful for inhibiting *Clostridium perfringens* in the chicks (La Ragione et al., 2004). *Prevotella* with the characteristic of decomposing starch and plant polysaccharides has strong catabolism of mucin (Gao et al., 2022). This changed abundance of microbiota reflected the dietary supplementation of AB and RBC at the dose of 5 and 10 g/kg, respectively, played a function in modulating the microbiota.

## Conclusion

In this study, the comparative effect of AB and RBC doses on weaning piglets was explored. The RBC1 supplementation at the dose of 5 g/kg improved the G/F and ATTD of DM of weaned piglets than the CON diet, while 10 g/kg of RBC improved the G/F and ADG than CON and AB diet; it also improved the ATTD of DM, N, and GE than the AB diet. These improved indicators may result in elevating the intestinal morphology of the jejunum and ileum and antioxidant properties in the serum (GSH and SOD) of weaning piglets by RBC. Meanwhile, AB supplementation also elevated the digestibility of DM, N, and GE, and the ileum intestinal morphology of weaning piglets than the controls. Additionally, the supplementation of AB, RBC1, and RBC2 modulated the microbiota in the colon but the RBC2 had a more profound influence on improving the α diversity than the controls. Taken together, the dietary RBC at the dose of 10 g/kg achieved a better effect on the weaning piglets than AB; thus, 10 g/kg of RBC could be an alternative to AB.

## Data availability statement

All standard sequence format (.fastq) files generated by Illumina Miseq containing all raw sequence reads have been deposited at the National Center for Biotechnology Information (NCBI) Sequence Read Archive (SRA) (BioProject accession number: PRJNA864102).

## Ethics statement

This experiment was conducted at the experiment base in Ya'an (Sichuan, China). All experimental and animal management procedures conformed to the Animal Care and Use Committee of Sichuan Agricultural University (Sichuan, China), and followed animal protection law (approval number: 20160125).

## Author contributions

JL designed the experiment, provided the funds, and revised the manuscript. QZ analyzed the data and wrote the manuscript. ZL performed the animal trial and determination of indices. XY helped to analyze indices. YL, SX, and YZ helped to design this experiment. ZF, BF, LC, and DW helped to revise the final manuscript. SL provided guide in animal trial. All authors approved the final manuscript.

## Conflict of interest

Author SL was employed by Guangzhou Prosyn Biological Technology Feed CO., LTD. The remaining authors declare that the research was conducted in the absence of any commercial or financial relationships that could be construed as a potential conflict of interest.

## Publisher's note

All claims expressed in this article are solely those of the authors and do not necessarily represent those of their affiliated organizations, or those of the publisher, the editors and the reviewers. Any product that may be evaluated in this article, or claim that may be made by its manufacturer, is not guaranteed or endorsed by the publisher.

## References

[B1] Ayiku S. Shen J. F. Tan B. P. Dong X. H. Liu H. Y. (2020). Effects of dietary yeast culture on shrimp growth, immune response, intestinal health and disease resistance against Vibrio harveyi. Fish Shellfish Immun. 102, 286–295. 10.1016/j.fsi.2020.04.036 32334129

[B2] Bao X. Wu J. (2021). Impact of food-derived bioactive peptides on gut function and health. Food Res. Int. 147, 110485. 10.1016/j.foodres.2021.110485 34399481

[B3] Berger K. Burleigh S. Lindahl M. Bhattacharya A. Patil P. St?lbrand H.. (2021). Xylooligosaccharides increase bifidobacteria and lachnospiraceae in mice on a high-fat diet, with a concomitant increase in short-chain fatty acids, especially butyric acid. J. Agr. Food Chem. 69, 3617–3625. 10.1021/acs.jafc.0c0627910.1021/acs.jafc.0c06279 33724030 PMC8041301

[B4] Bruininx E. M. A. M. Van Der Peet-Schwering C. M. C. Schrama J. W. Vereijken P. F. G. Vesseur P. C. Everts H.. (2001). Individually measured feed intake characteristics and growth performance of group-housed weanling pigs: effects of sex, initial body weight, and body weight distribution within groups. J. Anim. Sci.79, 301–308. 10.2527/2001.792301x 11219437

[B5] Campbell J. M. Crenshaw J. D. Polo J. (2013). The biological stress of early weaned piglets. J. Anim. Sci. Biotechno. 4, 1–4. 10.1186/2049-1891-4-19 23631414 PMC3651348

[B6] Caporaso J. G. Kuczynski J. Stombaugh J. Bittinger K. Bushman F. D. Costello E. K.. (2010). QIIME allows analysis of high-throughput community sequencing data. Nat. Methods 7, 335–336. 10.1038/nmeth.f.303 20383131 PMC3156573

[B7] Castillo M. Martínorúe S. M. Taylorpickard J. A. Pérez J. F. Gasa J. (2008). Use of mannanoligosaccharides and zinc chelate as growth promoters and diarrhea preventative in weaning pigs: effects on microbiota and gut function. J. Anim. Sci. 86, 94–101. 10.2527/jas.2005-686 17911238

[B8] Cui K. Diao Q. Zhang N. (2019). Effects of dietary supplementation with Bacillus subtilis and yeast culture on growth performance, nutrient digestibility, serum indices and faeces microbiota of weaned piglets. J. Anim. Feed. Sci. 28, 328–336. 10.22358/jafs/114238/2019

[B9] Dávila-Ramírez J. L. Carvajal-Nolazco M. R. López-Millanes M. J. González-Ríos H. Celaya-Michel H. Sosa-Castañeda J.. (2020). Effect of yeast culture (*Saccharomyces cerevisiae*) supplementation on growth performance, blood metabolites, carcass traits, quality, and sensorial traits of meat from pigs under heat stress. J. Anim. Feed. Sci. 267, 114573. 10.1016/j.anifeedsci.2020.114573

[B10] De Los Santos F. S. Donoghue A. M. Farnell M. B. Huff G. R. Huff W. E. Donoghue D. J. (2007). Gastrointestinal maturation is accelerated in turkey poults supplemented with a mannan-oligosaccharide yeast extract (Alphamune). Poult. Sci. 86, 921–930. 10.1093/ps/86.5.921 17435027

[B11] Dowarah R. Verma A. K. Agarwal N. (2017). The use of Lactobacillus as an alternative of antibiotic growth promoters in pigs: a review. Anim. Nutr. 3, 1–6. 10.1016/j.aninu.2016.11.002 29767055 PMC5941084

[B12] Elwan H. A. M. Elnesr S. S. Abdallah Y. Hamdy A. El-Bogdady A. H. (2018). Red yeast (*Phaffia rhodozyma*) as a source of astaxanthin and its impacts on productive performance and physiological responses of poultry. World Poultry Sci. J. 75, 273–284. 10.1017/S0043933919000187

[B13] Fattman C. L. Schaefer L. M. Oury T. D. (2003). Extracellular superoxide dismutase in biology and medicine. Free Radical Bio. Med. 35, 236–256. 10.1016/S0891-5849(03)00275-2 12885586

[B14] Fouhse J. M. Zijlstra R. T. Willing B. P. (2016). The role of gut microbiota in the health and disease of pigs. Anim. Front. 6, 30–36. 10.2527/af.2016-0031

[B15] Gang L. Lei Y. Martínez Y. Wenkai R. Hengjia N. Naif A. D.. (2017). Dietary saccharomyces cerevisiae cell wall extract supplementation alleviates oxidative stress and modulates serum amino acids profiles in weaned piglets. Oxid. Med. Cell. Longev. 3, 1–7. 10.1155/2017/3967439 28386308 PMC5366236

[B16] Gao J. Zhang H. J. Yu S. H. Wu S. G. Yoon I. Quigley J.. (2008). Effects of yeast culture in broiler diets on performance and immunomodulatory functions. Poult. Sci. 87, 1377–1384. 10.3382/ps.2007-00418 18577619

[B17] Gao X. Yu B. Yu J. Mao X. Huang Z. Luo Y.. (2022). Developmental profiling of dietary carbohydrate digestion in piglets. Front. Microbiol. 13, 896660. 10.3389/fmicb.2022.896660 35572714 PMC9100932

[B18] Ge Y. Huang K. Xie W. Xu C. Yao Q. Liu Y. (2021). Effects of *Rhodotorula mucilaginosa* on the immune function and gut microbiota of mice. Fronti. Fungal Biol. 2, 705696. 10.3389/ffunb.2021.705696 PMC10512290 37744147

[B19] Giang H. H. Viet T. Q. Ogle B. Lindberg J. E. (2012). Growth performance, digestibility, gut environment and health status in weaned piglets fed a diet supplemented with a complex of lactic acid bacteria alone or in combination with Bacillus subtilis and Saccharomyces boulardii. Livest. Sci. 143, 132–141. 10.1016/j.livsci.2011.09.00334557395

[B20] Haddad S. G. Goussous S. N. (2005). Effect of yeast culture supplementation on nutrient intake, digestibility and growth performance of Awassi lambs. Anim. Feed Sci. Tech. 118, 343–348 10.1016/j.anifeedsci.2004.10.003

[B21] Hampson D. J. (1986). Alterations in piglet small intestinal structure at weaning. Res. Vet. Sci. 40, 32–40. 10.1016/S0034-5288(18)30482-X 3704321

[B22] Hardo T. Brotosudarmo P. Limantara L. Setiyono E. (2020). Review article structures of astaxanthin and their consequences for therapeutic application. Int. J. Food Sci. 10, 1–16. 10.1155/2020/2156582 32775406 PMC7391096

[B23] He W. Gao Y. Guo Z. Yang Z. Wang X. Liu H.. (2021). Effects of fermented wheat bran and yeast culture on growth performance, immunity, and intestinal microflora in growing-finishing pigs. J. Anim. Sci. 9, skab308. 10.1093/jas/skab308 34687291 PMC8601129

[B24] Hu J. Lu W. Wang C. Zhu R. Qiao J. (2008). Characteristics of solid-state fermented feed and its effects on performance and nutrient digestibility in growing-finishing pigs. Asian. Austral. J. Anim. 21, 1635–1641. 10.5713/ajas.2008.80032

[B25] Hu R. He Z. Liu M. Tan J. Zhang H. Hou D. X. (2020). Dietary protocatechuic acid ameliorates inflammation and up-regulates intestinal tight junction proteins by modulating gut microbiota in LPS-challenged piglets. J. Anim. Sci. Biotechn. 11, 1–12. 10.1186/s40104-020-00492-9 32944233 PMC7487840

[B26] Hu Y. Dun Y. Li S. Zhang D. Peng N. Zhao S.. (2015). Dietary Enterococcus faecalis LAB31 improves growth performance, reduces diarrhea, and increases fecal Lactobacillus number of weaned piglets. PLoS ONE 10, e0116635. 10.1371/journal.pone.0116635 25617897 PMC4305361

[B27] La Ragione R. M. Narbad A. Gasson M. J. Woodward M. J. (2004). *In vivo* characterization of *Lactobacillus johnsonii* FI9785 for use as a defined competitive exclusion agent against bacterial pathogens in poultry. Lett. Appl. Microbiol. 38, 197–205. 10.1111/j.1472-765X.2004.01474.x 14962040

[B28] Lee D. J. Liu X. Sun H. Y. Park J. W. Kim I. H. (2018). Effects of yeast culture (*Saccharomyces cerevisiae*) supplementation on growth performance, fecal score, and nutrient digestibility of weaning pigs. J. Anim. Sci. 96, 48–49. 10.1093/jas/sky073.091

[B29] Lei Y. Kim I. H. (2014). Effect of *Phaffia rhodozyma* on performance, nutrient digestibility, blood characteristics, and meat quality in finishing pigs. J. Anim. Sci. 92, 171–176. 10.2527/jas.2013-6749 24352965

[B30] Li J. Sun W. Ramaswamy H. S. Yu Y. Zhu S. Wang J.. (2017). High pressure extraction of astaxanthin from shrimp waste (Penaeus Vannamei Boone): effect on yield and antioxidant activity. J. Food. Process. Eng. 40, e12353. 10.1111/jfpe.12353

[B31] Lin Y. Zhao W. Shi Z. D. Gu H. R. Zhang X. T. Ji X.. (2017). Accumulation of antibiotics and heavy metals in meat duck deep litter and their role in persistence of antibiotic-resistant *Escherichia coli* in different flocks on one duck farm. Poult. Sci. 96, 997–1006. 10.3382/ps/pew368 27744296

[B32] Long S. F. Xu Y. T. Pan L. Wang Q. Q. Wang C. L. Wu J. Y.. (2018). Mixed organic acids as antibiotic substitutes improve performance, serum immunity, intestinal morphology and microbiota for weaned piglets. Anim. Feed. Sci. Tech. 235, 23–32. 10.1016/j.anifeedsci.2017.08.018

[B33] Ma J. Piao X. Shang Q. Long S. Liu S. Mahfuz S. (2021). Mixed organic acids as an alternative to antibiotics improve serum biochemical parameters and intestinal health of weaned piglets. Anim. Nutr. 7, 737–749. 10.1016/j.aninu.2020.11.018 34466678 PMC8379140

[B34] Matrosova I. V. Politaeva A. A. (2021). Red yeast rhodotorula benthica – substitute feed base for echinoderms in factory cultivation. E3S Web of Conf. 265, 05010. 10.1051/e3sconf/202126505010

[B35] Montagne L. Cavaney F. S. Hampson D. J. Lallés J. P. Pluske J. R. (2004). Effect of diet composition on postweaning colibacillosis in piglets. J. Anim. Sci. 82, 2364–2374. 10.2527/2004.8282364x 15318736

[B36] Neuman H. Forsythe P. Uzan A. Avni O. Koren O. (2018). Antibiotics in early life: dysbiosis and the damage done. FEMS Microbiol. Rev. 42, 489–499. 10.1093/femsre/fuy018 29945240

[B37] NRC (2012). Nutrient Requirements of Swine. 11th rev. Edn. Washington, DC: Natl. Acad. Press.

[B38] Oh J. K. Vasquez R. Kim S. H. Hwang I. C. Song J. H. Park J. H.. (2021). Multispecies probiotics alter fecal short-chain fatty acids and lactate levels in weaned pigs by modulating gut microbiota. J. Anim. Sci. Tech. 63, 1142–1158. 10.5187/jast.2021.e94 34796353 PMC8564300

[B39] Oliver W. T. Wells J. E. (2013). Lysozyme as an alternative to antibiotics improves growth performance and small intestinal morphology in nursery pigs. J. Anim. Sci. 91, 3129–3136. 10.2527/jas.2012-5782 23572262

[B40] Pluske J. R. Hampson D. J. Williams I. H. (1997). Factors influencing the structure and function of the small intestine in the weaned pig: a review. Livest. Prod. Sci. 51, 215–236. 10.1016/S0301-6226(97)00057-2

[B41] Pluske J. R. Williams I. H. Aherne F. X. (1996). Villous height and crypt depth in piglets in response to increases in the intake of cows' milk after weaning. Anim. Sci. 62, 145–158. 10.1017/S1357729800014429

[B42] Primec M. Klemenak M. Di Gioia D. Aloisio I. Cionci N. B. Quagliariello A.. (2019). Clinical intervention using Bifidobacterium strains in celiac disease children reveals novel microbial modulators of TNF-α and short-chain fatty acids. Clin. Nutr. 38, 1373–1381. 10.1016/j.clnu.2018.06.931 29960810

[B43] Reisinger N. Ganner A. Masching S. Schatzmayr G. Applegate T. J. (2012). Efficacy of a yeast derivative on broiler performance, intestinalmorphology and blood profile. Livest. Sci.143, 195–200. 10.1016/j.livsci.2011.09.013

[B44] Ren H. Su X. Bai H. Yang Y. Wang H. Dan Z. (2019). Specific enrichment of microbes and increased ruminal propionate production: the potential mechanism underlying the high energy efficiency of Holstein heifers fed steam-flaked corn. Amb. Express. 9, 1–11. 10.1186/s13568-019-0937-8 31884565 PMC6935382

[B45] Ryazanov V. A. Duskaev G. K. Rakhmatullin S. G. Miroshnikov I. S. Miroshnikova K. P. Inchagova K. S. (2021). Application of new technologies to assess the effectiveness of feed materials for ruminants. IOP Conf. Ser. Earth Environ. Sci. 624, 012049. 10.1088/1755-1315/624/1/012049

[B46] Saied J. M. Al-Jabary Q. H. Thalij K. M. (2011). Effect of dietary supplement yeast culture on production performance and hematological parameters in broiler chicks. Int. J. Poult. Sci. 10, 376–380. 10.3923/ijps.2011.376.380

[B47] Segata N. Izard J. Waldron L. Gevers D. Miropolsky L. Garrett W. S.. (2011). Metagenomic biomarker discovery and explanation. Genome Biol. 12, 1–18. 10.1186/gb-2011-12-6-r60 21702898 PMC3218848

[B48] Shuai C. Bie T. Xia Y. Liao S. Wang M. Jie Y.. (2019). Effects of dietary gamma-aminobutyric acid supplementation on the intestinal functions in weaning piglets. Food Funct. 10, 366–378. 10.1039/C8FO02161A 30601517

[B49] Sims M. D. Dawson K. A. Newman K. E. Spring P. Hoogell D. M. (2004). Effects of dietary mannan oligosaccharide, bacitracin methylene disalicylate, or both on the live performance and intestinal microbiology of turkeys. Poult. Sci. 83, 1148–1154. 10.1093/ps/83.7.1148 15285506

[B50] Song B. Wu T. You P. Wang H. Burke J. L. Kang K.. (2021). Dietary supplementation of yeast culture into pelleted total mixed rations improves the growth performance of fattening lambs. Front. Vet. Sci. 8, 387. 10.3389/fvets.2021.657816 34055948 PMC8149762

[B51] Sun J. Li M. Tang Z. Zhang X. Chen J. Sun Z. (2020). Effects of Rhodotorula mucilaginosa fermentation product on the laying performance, egg quality, jejunal mucosal morphology and intestinal microbiota of hens. J. Appl. Microbiol. 128, 54–64. 10.1111/jam.14467 31562827

[B52] Tang Z. Yin Y. Zhang Y. Huang R. Sun Z. Li T.. (2008). Effects of dietary supplementation with an expressed fusion peptide bovine lactoferricin–lactoferrampin on performance, immune function and intestinal mucosal morphology in piglets weaned at age 21 d. Brit. J. Nutr. 101, 998–1005. 10.1017/S0007114508055633 18840311

[B53] Tian D. Xu X. Peng Q. Wen Z. Zhang Y. Wei C.. (2019). In vitro fermentation of arabinoxylan from oat (*Avena sativa* L.) by Pekin duck intestinal microbiota. 3 Biotech. 9, 1–12. 10.1007/s13205-019-1571-5 30729078 PMC6349265

[B54] Upadhaya S. D. Lee K. Y. Serpunja S. Song T. H. Kim I. H. (2018). Growth performance, nutrient digestibility, fecal microbiota and fecal noxious gas emission in weaning pigs fed high and low density diet with and without protected organic acid blends. Anim. Feed Sci. Tech. 239, 1–8. 10.1016/j.anifeedsci.2017.12.013

[B55] Wang C. Y. Liu S. Xie X. N. Tan Z. R. (2017). Regulation profile of the intestinal peptide transporter 1 (PepT1). Drug Des. Dev. Ther 11, 3511. 10.2147/DDDT.S151725 29263649 PMC5726373

[B56] Wang J. H. Zhao L. Q. Liu J. F. Wang H. Xiao S. (2015). Effect of potential probiotic *Rhodotorula benthica* D30 on the growth performance, digestive enzyme activity and immunity in juvenile sea cucumber *Apostichopus japonicus*. Fish Shellfish Immun. 43, 330–336. 10.1016/j.fsi.2014.12.028 25592878

[B57] Wang L. Xie J. Wu W. Li B. Ou J. (2018). Excellent microbial cultivation for astaxanthin production and its extraction by *Rhodotorula benthica*. Med. Res. 2, 180015. 10.21127/yaoyimr20180015

[B58] Wang T. Teng K. Liu Y. Shi W. Zhang J. Dong E.. (2019). *Lactobacillus plantarum* PFM 105 promotes intestinal development through modulation of gut microbiota in weaning piglets. Front. Microbiol. 10, 90. 10.3389/fmicb.2019.00090 30804899 PMC6371750

[B59] Yuan S. B. Chen D. W. Zhang K. Y. Yu B. (2007). Effects of oxidative stress on growth performance, nutrient digestibilities and activities of antioxidative enzymes of weanling pigs. Asian. Austral. J. Anim. 20, 1600–1605. 10.5713/ajas.2007.1600

[B60] Yun L. Wang W. Li Y. Xie M. Chen T. Hu C.. (2021). Potential application values of a marine red yeast, Rhodosporidiums sphaerocarpum YLY01, in aquaculture and tail water treatment assessed by the removal of ammonia nitrogen, the inhibition to *Vibrio* spp., and nutrient composition. PLoS ONE. 16, e0246841. 10.1371/journal.pone.0246841 33592044 PMC7886173

[B61] Zeitz J. O. Kaltenböck S. Most E. Eder K. (2017). Antioxidant status and expression of inflammatory genes in gut and liver of piglets fed different dietary methionine concentrations. J. Anim. Physiolo. An. N. 101, 1166–1174. 10.1111/jpn.12633 28066942

[B62] Zeng P. L. Yan H. C. Wang X. Q. Zhang C. M. Zhu C. Shu G.. (2013). Effects of dietary lysine levels on apparent nutrient digestibility and serum amino acid absorption mode in growing pigs. Asian. Austral. J. Anim. 26, 1003. 10.5713/ajas.2012.12555 25049879 PMC4093494

[B63] Zhang A. W. Lee B. D. Lee S. K. Lee K. W. An G. H. Song K. B.. (2005). Effects of yeast (*Saccharomyces cerevisiae*) cell components on growth performance, meat quality, and ileal mucosa development of broiler chicks. Poultry Sci. 84, 1015–1021. 10.1093/ps/84.7.1015 16050118

[B64] Zhang D. Ji H. Liu H. Wang S. Wang J. Wang Y. (2016). Changes in the diversity and composition of gut microbiota of weaned piglets after oral administration of Lactobacillus or an antibiotic. Appl. Microbiol. Biot. 100, 10081–10093. 10.1007/s00253-016-7845-5 27757509

[B65] Zhang J. Y. Park J. W. Kim I. H. (2019). Effect of supplementation with brewer's yeast hydrolysate on growth performance, nutrients digestibility, blood profiles and meat quality in growing to finishing pigs. Asian. Austral. J. Anim. 32, 1565. 10.5713/ajas.18.0837 31011001 PMC6718907

[B66] Zhang L. Loh K. C. Dai Y. Tong Y. W. (2020). Acidogenic fermentation of food waste for production of volatile fatty acids: bacterial community analysis and semi-continuous operation. Waste Manage. 109, 75–84. 10.1016/j.wasman.2020.04.052 32388405

[B67] Zhang Q. Li J. Cao M. Li Y. Zhuo Y. Fang Z.. (2020). Dietary supplementation of Bacillus subtilis PB6 improves sow reproductive performance and reduces piglet birth intervals. Anim. Nutr. 6, 278–287. 10.1016/j.aninu.2020.04.002 33005761 PMC7503085

