# Supplementary material for: Rhodotorula benthica culture as an alternative to antibiotics improves growth performance by improving nutrients digestibility and intestinal morphology, and modulating gut microbiota of weaned piglets
Source: Front Microbiol. 2022 Sep 2;13:964531. doi: 10.3389/fmicb.2022.964531 (PMC9479635; doi:10.3389/fmicb.2022.964531)
Supplement: Supplementary file 1 [file Data_Sheet_1.docx]

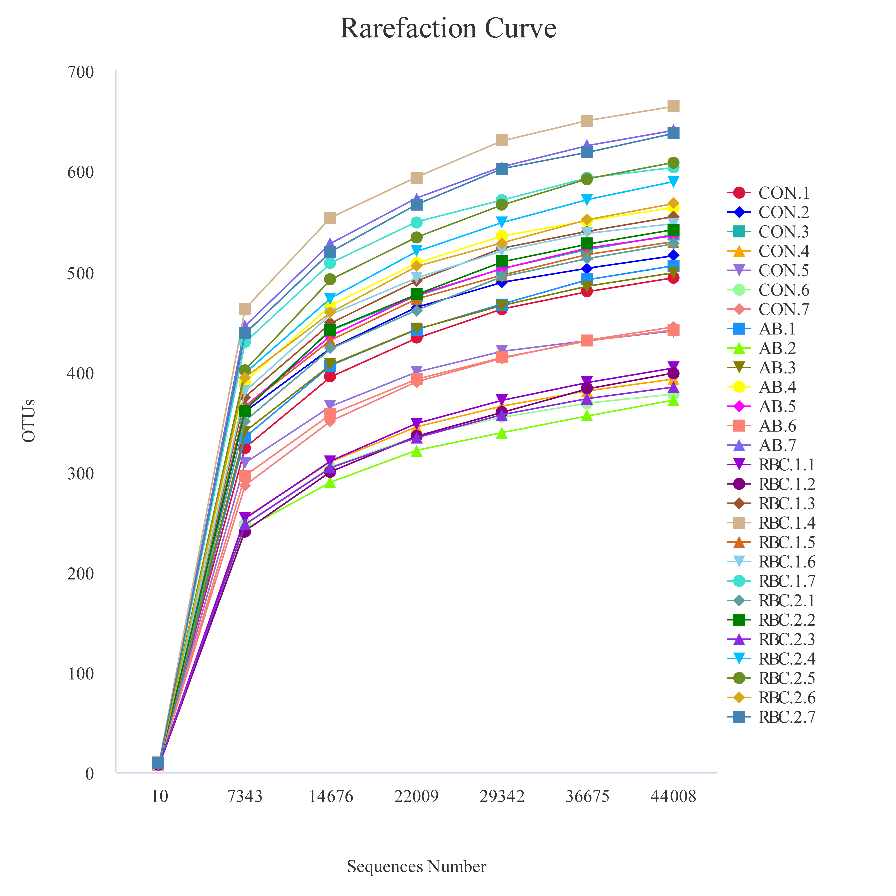


Figure S1. Sequencing depth at the OTUs level in all samples.

| Table S1 The effect of RBC and AB on the effect of the relative abundance of microbiota used Metastat analysis at the phylum level in the colonic digesta | | | | | | | | | | |
| --- | --- | --- | --- | --- | --- | --- | --- | --- | --- | --- |
| Items | CON | AB | RBC1 | RBC2 | *P*-value | | | | | |
|  |  |  |  |  | CON vs  AB | CON vs  RBC1 | CON vs  RBC2 | AB vs  RBC1 | AB vs  RBC2 | RBC1 vs  RBC2 |
| *Firmicutes* | 0.448 ± 0.016 | 0.553±0.033 | 0.509±0.042 | 0.573± 0.018 | 0.015 | 0.154 | 0.005 | 0.530 | 0.698 | 0.153 |
| *Bacteroidota* | 0.284±0.018 | 0.287±0.034 | 0.320±0.026 | 0.350±0.030 | 0.906 | 0.509 | 0.047 | 0.568 | 0.132 | 0.281 |
| *Proteobacteria* | 0.100±0.033 | 0.040±0.009 | 0.050±0.019 | 0.022±0.008 | 0.073 | 0.189 | 0.019 | 0.732 | 0.113 | 0.203 |
| *Unidentified_Bacteria* | 0.083±0.018 | 0.072±0.012 | 0.079±0.018 | 0.084±0.008 | 0.666 | 0.846 | 0.898 | 0.806 | 0.470 | 0.788 |
| *Desulfobacterota* | 0.003±0.000 | 0.012±0.008 | 0.005±0.001 | 0.005±0.001 | 0.260 | 0.293 | 0.225 | 0.460 | 0.566 | 0.861 |
| *Cyanobacteria* | 0.004±0.002 | 0.002±0.000 | 0.012±0.006 | 0.002±0.000 | 0.550 | 0.246 | 0.347 | 0.109 | 0.325 | 0.110 |
| *Actinobacteriota* | 0.001±0.000 | 0.008±0.006 | 0.001±8.48E | 0.002±0.000 | 0.284 | 0.074 | 0.763 | 0.221 | 0.142 | 0.154 |
| *Spirochaetota* | 0.002±0.001 | 0.004±0.002 | 0.009±0.006 | 0.006±0.004 | 0.199 | 0.237 | 0.273 | 0.613 | 0.144 | 0.753 |
| *Euryarchaeota* | 0.003±0.001 | 0.006±0.003 | 0.002±0.001 | 0.011±0.004 | 0.180 | 0.357 | 0.036 | 0.045 | 0.447 | 0.015 |
| *Campylobacterota* | 0.003±0.002 | 0.003±0.001 | 0.006±0.004 | 0.001±0.000 | 0.415 | 0.278 | 0.733 | 0.589 | 0.225 | 0.871 |

| Table S2The effect of RBC and AB on the effect of the relative abundance of microbiota used Metastat analysis at the genus level in the colonic digesta | | | | | | | | | | |
| --- | --- | --- | --- | --- | --- | --- | --- | --- | --- | --- |
| Items | CON | AB | RBC1 | RBC2 | *P*-value | | | | | |
|  |  |  |  |  | CON vs  AB | CON vs  RBC1 | CON vs  RBC2 | AB vs  RBC1 | AB vs  RBC2 | RBC1 vs  RBC2 |
| *Lactobacillus* | 0.034±0.012 | 0.050±0.025 | 0.116±0.042 | 0.070±0.015 | 0.678 | 0.063 | 0.060 | 0.189 | 0.613 | 0.355 |
| *Prevotella_9* | 0.105±0.014 | 0.010±0.021 | 0.105±0.019 | 0.077±0.029 | 0.862 | 0.967 | 0.478 | 0.899 | 0.655 | 0.518 |
| *Mitsuokella* | 0.059±0.011 | 0.038±0.013 | 0.054±0.028 | 0.043±0.026 | 0.276 | 0.879 | 0.663 | 0.719 | 0.909 | 0.827 |
| *Succinivibrio* | 0.091±0.031 | 0.031±0.008 | 0.044±0.020 | 0.020±0.007 | 0.052 | 0.221 | 0.023 | 0.648 | 0.290 | 0.292 |
| *Dialister* | 0.045±0.018 | 0.060±0.021 | 0.025±0.016 | 0.028±0.015 | 0.693 | 0.459 | 0.561 | 0.200 | 0.238 | 0.886 |
| *Prevotella_7* | 0.041±0.023 | 0.022±0.012 | 0.002±0.001 | 0.004±0.001 | 0.571 | 0.101 | 0.104 | 0.101 | 0.122 | 0.617 |
| *Limosilactobacillus* | 0.015±0.089 | 0.021±0.010 | 0.028±0.007 | 0.041±0.016 | 0.759 | 0.276 | 0.172 | 0.667 | 0.358 | 0.545 |
| *Prevotellaceae_NK3B31_group* | 0.017±0.008 | 0.031±0.015 | 0.042±0.015 | 0.019±0.005 | 0.528 | 0.138 | 0.819 | 0.687 | 0.602 | 0.151 |
| *Acidaminococcus* | 0.024±0.004 | 0.037±0.014 | 0.009±0.002 | 0.013±0.003 | 0.495 | 0.003 | 0.029 | 0.045 | 0.088 | 0.330 |
| *Rikenellaceae_RC9_gut_group* | 0.029±0.014 | 0.032±0.010 | 0.026±0.006 | 0.059±0.013 | 0.878 | 0.870 | 0.128 | 0.662 | 0.099 | 0.027 |
| *CAG-873* | 0.020±0.015 | 0.002±0.002 | 0.001±0.001 | 0.005±0.002 | 0.201 | 0.206 | 0.336 | 0.837 | 0.236 | 0.110 |
| *Megasphaera* | 0.043±0.006 | 0.052±0.009 | 0.035±0.006 | 0.043±0.010 | 0.519 | 0.582 | 0.970 | 0.253 | 0.654 | 0.658 |
| *Lachnospiraceae_XPB1014_group* | 6.48E±1.88E | 0.002±0.002 | 0.000±0.000 | 0.010±0.009 | 0.355 | 0.176 | 0.286 | 0.431 | 0.487 | 0.303 |
| *Prevotella* | 0.030±0.011 | 0.013±0.004 | 0.031±0.00 | 0.020±0.004 | 0.145 | 0.914 | 0.461 | 0.011 | 0.305 | 0.101 |
| *Christensenellaceae_R-7_group* | 0.007±0.003 | 0.008±0.003 | 0.006±0.002 | 0.022±0.010 | 0.837 | 0.890 | 0.126 | 0.742 | 0.154 | 0.106 |
| *g__UCG-002* | 0.031±0.007 | 0.022±0.004 | 0017±0.005 | 0.046±0.010 | 0.297 | 0.137 | 0.237 | 0.569 | 0.018 | 0.011 |
| *Anaerovibrio* | 0.017±0.009 | 0.007±0.004 | 0.018±0.006 | 0.003±0.001 | 0.334 | 0.939 | 0.093 | 0.110 | 0.280 | 0.008 |
| *NK4A214_group* | 0.010±0.003 | 0.008±0.002 | 0.009±0.002 | 0.021±0.008 | 0.727 | 0.870 | 0.195 | 0.811 | 0.108 | 0.154 |
| *Erysipelotrichaceae_UCG-002* | 0.0.009009± | 8.10E±0.002 | 0.000±0.000 | 0.003±0.002 | 0.397 | 0.380 | 0.583 | 0.690 | 0.378 | 0.394 |
| *Agathobacter* | 0.0145±0.005 | 0.019±0.004 | 0.020±0.004 | 0.020±0.007 | 0.690 | 0.502 | 0.668 | 0.852 | 0.921 | 0.972 |
| *Prevotellaceae_UCG-003* | 0.009±0.009 | 0.008±0.003 | 0.022±0.006 | 0.016±0.004 | 0.824 | 0.041 | 0.123 | 0.030 | 0.099 | 0.502 |
| *Alloprevotella* | 0.014±0.007 | 0.008±0.003 | 0.020±0.005 | 0.004±0.002 | 0.522 | 0.495 | 0.199 | 0.019 | 0.448 | 0.002 |
| *Ruminococcus* | 0.007±0.003 | 0.021±0.008 | 0.012±0.004 | 0.007±0.002 | 0.079 | 0.275 | 0.860 | 0.411 | 0.077 | 0.281 |
| *Lachnospiraceae_NK4A136_group* | 0.008±0.000 | 0.005±0.002 | 0.004±0.003 | 0.008±0.007 | 0.061 | 0.255 | 0.338 | 0.863 | 0.792 | 0.698 |
| *Olsenella* | 0.001±0.00 | 0.007±0.006 | 0.000±9.19E | 0.001±0.001 | 0.400 | 0.064 | 0.291 | 0.316 | 0.420 | 0.268 |
| *Treponema* | 0.002±0.000 | 0.004±0.002 | 0.009±0.006 | 0.004±0.002 | 0.233 | 0.279 | 0.068 | 0.609 | 0.861 | 0.514 |
| *Megamonas* | 0.003±0.002 | 0.009±0.005 | 0.000±0.000 | 0.000±0.000 | 0.332 | 0.092 | 0.844 | 0.089 | 0.081 | 0.729 |
| *Streptococcus* | 0.006±0.003 | 0.006±0.003 | 0.004±0.003 | 0.007±0.005 | 0.981 | 0.690 | 0.428 | 0.742 | 0.866 | 0.666 |
| *Holdemanella* | 0.002±0.000 | 0.007±0.005 | 0.001±0.000 | 0.001±0.000 | 0.482 | 0.070 | 0.919 | 0.341 | 0.380 | 0.776 |
